# Prognostic Value of *PCSK9* Levels in Premenopausal Women at Risk of Breast Cancer—Evidence from a 17-Year Follow-Up Study

**DOI:** 10.3390/cancers16071411

**Published:** 2024-04-04

**Authors:** Massimiliano Ruscica, Chiara Macchi, Sara Gandini, Debora Macis, Aliana Guerrieri-Gonzaga, Valentina Aristarco, Davide Serrano, Matteo Lazzeroni, Alessandra Stefania Rizzuto, Aurora Gaeta, Alberto Corsini, Marcella Gulisano, Harriet Johansson, Bernardo Bonanni

**Affiliations:** 1 Department of Pharmacological and Biomolecular Sciences “Rodolfo Paoletti”, Università degli Studi di Milano, 20122 Milan, Italy; massimiliano.ruscica@unimi.it (M.R.); chiara.macchi@unimi.it (C.M.); alberto.corsini@unimi.it (A.C.); 2 Department of Cardio-Thoracic-Vascular Diseases, Foundation IRCCS Cà Granda Ospedale Maggiore Policlinico, 20122 Milan, Italy; 3 Department of Experimental Oncology, IEO, European Institute of Oncology IRCCS, 20141 Milan, Italy; sara.gandini@ieo.it (S.G.); aurora.gaeta@ieo.it (A.G.); 4 Division of Cancer Prevention and Genetics, IEO, European Institute of Oncology IRCCS, 20141 Milan, Italy; debora.macis@ieo.it (D.M.); aliana.guerrierigonzaga@ieo.it (A.G.-G.); valentina.aristarco@ieo.it (V.A.); davide.serrano@ieo.it (D.S.); matteo.lazzeroni@ieo.it (M.L.); bernardo.bonanni@ieo.it (B.B.); 5 Department of Clinical Sciences and Community Health, Università degli Studi di Milano, 20122 Milan, Italy; alessandra.rizzuto@unimi.it; 6 Department of Statistics and Quantitative Methods, University of Milan-Bicocca, 20126 Milan, Italy; 7 Medical Oncology, Ospedale S. Bortolo, 36100 Vicenza, Italy; marcella.gulisano@gmail.com

**Keywords:** biomarker, breast cancer, *PCSK9*, lipids

## Abstract

**Simple Summary:**

Breast cancer is recognized as the most common cancer within the female population. In this context, cholesterol is recognized as a vital component for the proliferation and survival of cancer cells. These cells primarily acquire cholesterol through the receptor-mediated uptake from external sources, including low-density lipoprotein (LDL), utilizing the endocytosis pathway. Thus, this observational study aimed to test the effectiveness of proprotein convertase subtilisin/kexin type 9 (*PCSK9*), one of the key regulators of cholesterol levels, as a prognostic biomarker in cancer onset. Although *PCSK9* correlated with lipid parameters (e.g., cholesterol, LDL) and with 17 β-estradiol, our results do not portend *PCSK9* is a prognostic biomarker, at least, in the context of breast neoplastic events.

**Abstract:**

Background and aim: The involvement of cholesterol in cancer development remains a topic of debate, and its association with breast cancer has yet to be consistently demonstrated. Considering that circulating cholesterol levels depend on several concomitant processes, we tested the liability of plasma levels of proprotein convertase subtilisin/kexin type 9 (*PCSK9*), one of the key regulators of cholesterol levels, as a prognostic biomarker in the context of breast neoplastic events. Methods: Within a prospective randomized breast cancer prevention trial we measured baseline plasma levels of *PCSK9*. A total of 235 at-risk premenopausal women were randomized and followed up for 17 years. Participants enrolled in this placebo-controlled, phase II, double-blind trial were randomly assigned to receive either tamoxifen 5 mg/d or fenretinide 200 mg/d, both agents, or placebo for 2 years. The associations with breast cancer events were evaluated through competing risk and Cox regression survival models, adjusted for randomization strata (5-year Gail risk ≥ 1.3% vs. intraepithelial neoplasia or small invasive breast cancer of favorable prognosis), age, and treatment allocation. *PCSK9* associations with biomarkers linked to breast cancer risk were assessed on blood samples collected at baseline. Results: The plasmatic *PCSK9* median and interquartile range were 207 ng/mL and 170–252 ng/mL, respectively. Over a median follow-up period of 17 years and 89 breast neoplastic events, disease-free survival curves showed a hazard ratio of 1.002 (95% CI: 0.999–1.005, *p* = 0.22) for women with *PCSK9* plasma levels ≥ 207 ng/mL compared to women with levels below 207 ng/mL. No differences between randomization strata were observed. We found a negative correlation between *PCSK9* and estradiol (r = −0.305), maintained even after partial adjustment for BMI and age (r = −0.287). Cholesterol (r = 0.266), LDL-C (r = 0.207), non-HDL-C (r = 0.246), remnant cholesterol (r = 0.233), and triglycerides (r = 0.233) also correlated with *PCSK9*. Conclusions: In premenopausal women at risk of early-stage breast cancer, *PCSK9* did not appear to have a role as a prognostic biomarker of breast neoplastic events. Larger studies are warranted investigating patients in different settings.

## 1. Introduction

Within the global female population, breast cancer stands out as the most prevalent form of cancer, representing 24% of newly diagnosed cancer cases and 15% of cancer-related deaths in 2018. It is expected that incident cases will increase by over 46% by the year 2040 [1]. In this context, although there is increasing evidence suggesting an association between cholesterol availability and cancer progression [2], the existence of a causal relationship between these two factors remains to be proven. Cholesterol affects cell proliferation, given its structural role in the local synthesis of steroid hormones and oxysterols, alongside the regulation of cell signaling [3]. It is also known as a pivotal component in various metabolic pathways, particularly in demanding anabolic processes such as cell growth and division, which are especially pertinent in tumor proliferation and metastasis [4]. Whilst Mendelian randomization studies have provided evidence supporting the association between genetically elevated low-density lipoprotein cholesterol (LDL-C) levels and an increased risk of breast cancer, observational studies have failed to report such a relationship [5,6]. Considering that proprotein convertase subtilisin/kexin type 9 (*PCSK9*) is one of the key regulators of cholesterol levels, evaluating its circulating levels could be of interest [7]. Initially identified as a neural apoptosis-regulated convertase 1 [8], *PCSK9* is the ninth member of the family of proprotein convertases. These proteins are synthesized as zymogens, inactive precursors chaperoned across the cell by their prodomains. *PCSK9*, which consists of 692 amino acids (aa), undergoes a single autocatalysis of the zymogen between aa Gln_152_ and Ser_153_ in the endoplasmic reticulum. Besides the prodomain, the 692 aa *PCSK9* protein contains a catalytic and a c-terminal domain [9]. Primarily expressed and secreted by the liver, *PCSK9* coordinates cholesterol metabolism by fostering the degradation of LDL receptors in the lysosomes [10]. *PCSK9* has also been described as being expressed in many other tissues, including the pancreatic and visceral adipose tissues as well [11].

Whilst most studies focus on its role in atherosclerosis, there is a growing interest in the involvement of *PCSK9* in cancer [12], particularly regarding its contribution to increased cholesterol levels and as a suppressor of the immune response. Indeed, *PCSK9* triggers the degradation of the Major Histocompatibility Complex 1 (MHC-I) receptor [13]. With the scope of exploring its role as a biomarker element, circulating plasma *PCSK9* levels were evaluated in 14 women with a benign breast condition, in 9 with stage 0, and in 23 with a stage III breast tumor. The latter group exhibited significantly increased levels compared to age-matched counterparts with a benign lesion [14]. Genetic studies have demonstrated that LDL-cholesterol-lowering variants in *PCSK9*, responsible for reduced levels of LDL, were associated with either a risk-reducing [15] or a neutral effect on breast cancer [16].

Given the current lack of clinical data assessing the employment of circulating *PCSK9* as a prognostic biomarker in the context of breast neoplastic events, we conducted a prospective analysis within a randomized chemoprevention trial for premenopausal women at risk of breast cancer. This was a 2 × 2 trial of low-dose tamoxifen, fenretinide, both agents, or placebo for a 2-year treatment duration, recruiting 235 women at risk of breast cancer who were followed up for over 17 years [17].

## 2. Materials and Methods

### 2.1. Participants

A total of 235 premenopausal women were enrolled in this phase II, double-blind, placebo-controlled 2 × 2 trial. Eligible participants were premenopausal healthy women with an increased 5-year Gail risk of 1.3% or greater (*n* = 54), or women with a previous diagnosis of intraepithelial neoplasia (IEN, *n* = 160) or small invasive cancer of a favorable prognosis (T1, *n* = 21). Participants were randomly assigned to receive either tamoxifen 5 mg/d or fenretinide 200 mg/d, both agents, or placebo for 2 years [18,19]. The enrollment took place between 1998 and 2002, and the treatments were completed in 2005. Participants were stratified according to the participating center (Milan/Vicenza) and breast cancer risk status (Gail risk versus previous diagnosis of breast carcinoma). Serum and plasma samples were collected at baseline before treatment started. All participants gave their written informed consent to participate in the study. The study was approved by the local institutional review boards.

### 2.2. Assay Methods

Fasting serum samples for circulating biomarker measurements were collected and stored at −80 °C until assay performance. Plasma concentrations of *PCSK9* were assessed by a commercial ELISA kit (R&D Systems, Minneapolis, MN, USA). Samples were diluted at 1:20 and incubated onto a microplate pre-coated with a monoclonal human-*PCSK9*-specific antibody. Sample concentrations were obtained by a four-parameter logistic curve fit, with a minimum detectable *PCSK9* concentration of 0.219 ng/mL [20]. Percentage mammographic density was centrally measured on analogue screen films or digital scans by a single trained radiologist, using a computer-assisted method [21].

Additionally, for the correlation analysis, we also included metabolic biomarkers that were already published, to assess their relationship with *PCSK9*. Briefly, serum concentrations of estradiol and insulin were determined by radioimmunoassay (RIA) kits purchased from Diagnostic Systems Laboratories (Webster, TX, USA). Serum concentrations of total cholesterol, high-density lipoprotein (HDL-C), LDL-C, triglycerides and glucose were determined in fresh samples by an enzymatic method with Cobas Integra 800 (Roche Diagnostics, Basel, Switzerland), as previously described [22]. Non-HDL-C was calculated as total cholesterol minus HDL-C. Remnant cholesterol was calculated as total cholesterol minus HDL-C minus LDL-C. Serum concentrations of C-reactive protein (CRP) were determined by a high-sensitivity assay using a two-site chemiluminescent enzyme immunometric assay (Diagnostic Products Corp, Los Angeles, CA, USA) for the IMMULITE automated analyzer. Plasma concentrations of leptin were measured using an RIA kit (Linco research, St Charles, MO, USA) and adiponectin was measured using a commercial enzyme-linked immunosorbent assay kit (R&D Systems, Minneapolis, MN, USA). HOMA was employed as a surrogate index of insulin sensitivity, i.e., [fasting insulinemia (mU/L) × glycemia (mmol/L)]/22.5] [23].

### 2.3. Statistical Analysis

We presented median values and interquartile ranges (IQRs) of serum biomarkers at baseline by risk stratum [(DIN, LIN, pT1mic or pT1a) vs. Gail]. Correlations among biomarkers were measured through Spearman correlation coefficients. Differences by groups were evaluated by Wilcoxon rank tests. We evaluated the association of *PCSK9* with the follow-up events, considering the biomarkers as continuous and categorical variables and with different cut-off points (median, and interquartile ranges). In addition, we presented the log-rank test to investigate the difference in the Kaplan–Meier curve by the median values of *PCSK9* and the hazard ratio with 95% confidence intervals from the multivariable Cox proportional hazard model, adjusted for age, risk strata, and trial arms. The distribution plots of *PCSK9* by risk groups is also presented. A two-tailed *p*-value < 0.05 was considered statistically significant. The statistical analyses were performed with R software, version 4.3.0.

## 3. Results

### 3.1. Baseline Patient Characteristics

Baseline patient characteristics and circulating biomarker results by risk category are depicted in Table 1. No inter-group differences between risk strata were found for age, BMI, and percentage mammographic density, as well as for the majority of circulating biomarkers, including *PCSK9*, total cholesterol, LDL-C, HDL-C, non-HDL-C, remnant cholesterol, and triglycerides. Conversely, plasma adiponectin concentrations were significantly higher in healthy women with a Gail risk ≥ 1.3% (12 vs. 9.8 µg/mL; *p* = 0.006) compared to women with a previous diagnosis of intraepithelial neoplasia. This latter risk category (IEN, T1) had statistically significantly higher insulin levels (14 vs. 12.3 µU/mL, *p* = 0.048) and HOMA-IR (3.05 vs. 2.65, *p* = 0.047) compared to the Gail risk group, without significant changes in fasting glucose.

**Figure 1:**
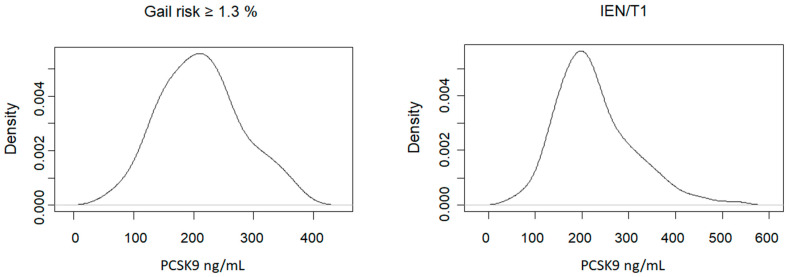
Distribution of fasting plasma concentrations of *PCSK9* levels in healthy women with an increased 5-year Gail risk versus women with intraepithelial neoplasia (IEN) or T1.

The overall distribution of plasma *PCSK9* levels according to risk groups is depicted in Figure 1.

### 3.2. Correlation Analyses

Correlations among biomarkers measured through Spearman correlation coefficients are reported in Table 2. Given the aim of the present study, it is worth considering that the strongest correlation we found was the negative one between *PCSK9* and 17-β estradiol (r = −0.294), which was maintained even after partial adjustment for BMI and age (r = −0.251), two risk factors for breast cancer. Conversely, the correlation coefficient for the relationship with percentage mammographic density, an important predictor of breast cancer risk, was negligible. Given the known role of adipose tissue in cancer initiation and progression, and considering that adiponectin was a risk biomarker for breast cancer in the same cohort [18], we assessed the relationship between *PCSK9* and the adipokines adiponectin and leptin. A poor correlation was found with leptin (r = 0.171), whereas that with adiponectin was negligible (r = 0.003). Considering that *PCSK9* is one of the key regulators of LDL-C homeostasis, the relationship with the lipid variables was assessed. The strongest correlations (all positive) were found with total cholesterol (r = 0.277), LDL-C (r = 0.217), non-HDL-C (r = 0.256), remnant cholesterol (r = 0.237), and triglycerides (r = 0.238). Eventually, the correlation coefficients for the relationship between plasma *PCSK9* and serumglucose or serum CRP were weak (r = 0.164 and r = 0.165, respectively).

### 3.3. Prognostic Value of Plasma Levels of PCSK9

**Figure 2:**
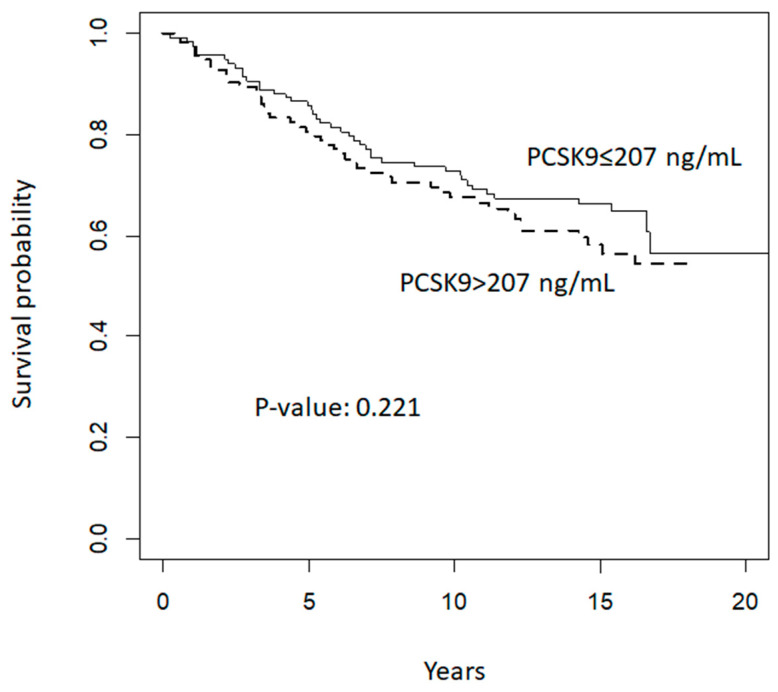
Disease-free survival according to median baseline *PCSK9* levels (ng/mL).

When exploring the prognostic value of circulating plasma levels of *PCSK9* on breast neoplastic events during follow up, we did not find any significant association between *PCSK9* and disease-free survival. Kaplan–Meier curves according to median baseline *PCSK9* plasma levels (i.e., below or equal to 207 ng/mL versus above 207 ng/mL) are presented in Figure 2. The median follow-up period was 17 years and the analysis included 77 breast neoplastic events (29 premalignant lesions and 48 invasive breast cancer lesions), and one sarcoma in the breast. Furthermore, we reported 11 events of other cancer types. The hazard ratio of events for women with *PCSK9* plasma levels above 207 ng/mL, adjusted for risk strata, age, and treatment arm allocation (tamoxifen plus placebo; fenretinide plus placebo; tamoxifen plus fenretinide; placebo plus placebo) compared to women with levels equal to or below 207 ng/mL was 1.002 (95% confidence interval: 0.999–1.005, *p* = 0.22).

We also evaluated the influence of other risk biomarkers, both as confounders and risk stratifiers, and different cut-off points for *PCSK9*, but the results did not change.

## 4. Discussion

The main result of the present study did not show any evidence of a role of circulating *PCSK9* as a prognostic biomarker of recurrent or new breast neoplastic events. This conclusion is based on long-term follow up of a median of 17 years, involving premenopausal women with a previous diagnosis of an intraepithelial neoplasia or microinvasive breast cancer, or healthy women at increased risk of neoplasia (5-year Gail risk ≥ 1.3%). To the best of our knowledge, this is the longest study of its kind to have been conducted so far. Indeed, only one recent study on a small sample size (*n* = 46) of women with stage III breast cancer has been conducted, reporting increasing levels of *PCSK9* with the severity of breast disease. However, no prognostic conclusions were made [14]. A pan-cancer analysis found that *PCSK9* expression was considerably higher in invasive breast carcinoma [24]. A large Mendelian randomization study including results from >400,000 participants showed that LDL-C variants mimicking *PCSK9* inhibitors were associated with a lower risk [15]. In line with this evidence, pharmacological inhibition of *PCSK9* improved breast cancer outcomes in BALB/c mice bearing 4T1 breast cancer [25]. In this scenario, it is worth mentioning that inhibition of *PCSK9* (evolocumab) is being tested in metastatic pancreatic cancer (NCT-04862260) and glioblastoma (NCT-04937413). Besides breast cancer, the involvement of *PCSK9* in cancer development has been described in different tissues [26]. In esophageal squamous cell carcinomas, *PCSK9* was highly expressed in cancerous tissues compared with normal esophageal tissues, and its expression was associated with a poorer prognosis [27]. A similar conclusion was reached in patients with head and neck squamous cell carcinoma, in whom a higher tissue protein *PCSK9* expression indicated not only a poorer prognosis but also a stemness-like phenotype and a higher infiltration and activity of CD8^+^ T lymphocytes. However, no information was reported on circulating *PCSK9* levels [28]. These findings suggest a paracrine activity of *PCSK9* within the tumor environment rather than an endocrine effect. Indeed, as reviewed elsewhere, several preclinical studies have hypothesized that inhibition of *PCSK9* through genetic depletion, monoclonal antibodies, nano-liposomal vaccination, or siRNA might effectively suppress tumor growth [26]. In hepatocellular carcinoma, *PCSK9* was found to promote cell growth by inhibiting cell apoptosis via the involvement of the Bax/Bcl-2/Caspase-9/Caspase-3 pathway [29]. *PCSK9* expression was described as being upregulated in colon cancer tissue versus corresponding adjacent normal tissue, and associated with tumors of a pathological grade. In colon cancer cell lines, *PCSK9* was shown to promote cell progression and metastasis by downregulating E-cadherin expression, inducing the colon cancer cell epithelial–mesenchymal transition process and activating PI3K/AKT signaling [30]. In human gastric cancer, through the upregulation of heat shock protein 70 in the MAPK signaling pathway, *PCSK9* promoted cell invasion and suppressed apoptosis. Increased *PCSK9* expression was related to tumor progression and poor survival [31]. *PCSK9* has also been found to be involved in the regulation of inflammation via a suppressor of cytokine signaling-3 (SOCS3) and signal transducer and activator of the transcription 3 (STAT3) pathway [32], as well as of cell proliferation and apoptosis [33].

Concerning the negative correlation between *PCSK9* and 17-β-estradiol, it is worth considering that the impact of reproductive hormones on the expression of *PCSK9* levels remains a field fraught with uncertainties. In line with our data, a cross-sectional study demonstrated that E2 correlates inversely with *PCSK9*, despite E2 therapy not influencing *PCSK9* levels [34]. In a cohort of 206 premenopausal females, the estrous cycle was strongly related to *PCSK9* levels, particularly with higher concentrations during the follicular phase compared to the ovulatory or luteal phases [35]. In 31 healthy women who underwent in vitro fertilization, the increased levels of endogenous estradiol, following the administration of follicle-stimulating hormone, led to a 14% reduction in *PCSK9* levels [36]. Other studies have shown that postmenopausal women exhibit significantly higher median *PCSK9* plasma levels compared to premenopausal women. Additionally, estrogen replacement in postmenopausal women is not associated with a difference in median *PCSK9* levels [37]. Furthermore, *PCSK9* levels are substantially elevated compared to non-pregnant age-matched women (493 vs. 290 ng/mL) [38]. Conversely, preclinical evidence displays that in apoE^−/−^ mice subjected to ovariectomy, exogenous estrogens cause *PCSK9* levels to increase [39]. On the other hand, although cholesterol is necessary for gonadal steroid hormone biosynthesis, there is no evidence that *PCSK9* can influence estrogen metabolism. Treatment with evolocumab did not alter the levels of estradiol, over a period of 52 weeks, in women with hypercholesterolemia [40]. These findings were confirmed in patients with type 2 diabetes and dyslipidemia who received evolocumab for 12 weeks [41].

Owing to the fact that metabolic risk factors (e.g., diet, obesity, lack of physical activity) are known common denominators in both cardiovascular diseases and breast cancer, the role of lipids in the latter disease context should not be ignored. Although a direct relationship between serum cholesterol parameters and incidence of hormonally driven cancer is still missing, we assessed whether *PCSK9* levels correlates with changes in lipids and found that *PCSK9* was positively correlated with an atherogenic pattern (i.e., LDL-C, non-HDL-C, and remnant cholesterol). Tumor cells display metabolic changes that are known to correlate with malignancy, such as the development of a lipogenic phenotype [42], leading to a dysregulated accumulation of cholesterol [43]. From the analysis of health insurance claims and health check-up data of more than 950,000 women in Japan, it was evident that women with LDL-C higher than 140 mg/mL had a modest but significantly increased risk of breast cancer vs. women with LDL-C levels lower than 140 mg/mL. Conversely, no associations were observed between HDL-C and triglycerides with breast cancer risk [44]. A meta-analysis of prospective studies concluded that there was no association between LDL-C and the risk of breast cancer, while a modest but statistically significant inverse association between total cholesterol, specifically HDL-C, and the risk of breast cancer was observed [5]. Mendelian randomization studies reported that genetically elevated plasma HDL-C and LDL-C levels due to a genetic cause were positively associated with breast cancer risk [45], whilst no such association was found in corresponding observational studies. Overall, this indicates that there is no association between elevated levels of HDL-C and LDL-C and the risk of breast cancer [6].

Considering the general relationship between adipokines and *PCSK9* [46,47], we sought to investigate said relationship for certain adipokines. While in in vitro models of hepatocytes, leptin caused the gene and protein expression of *PCSK9* to rise [47] and had no effect on plasma *PCSK9* in female ob/ob mice, leptin treatment reduced plasma *PCSK9* in female patients with lipodystrophy [48]. In fact, the positive association between *PCSK9* and leptin that we observed was weak and became negligible when the analysis was partially adjusted for BMI and age (r = 0.140) is in line with a previous observation demonstrating a positive association between these two variables only when the body mass index was <25 kg/m^2^ [47].

Overall, these results should be interpreted with caution. Firstly, this is an observational study, and an a priori sample size calculation was not performed to achieve an appropriate level of power. Despite quite a large sample size of premenopausal women, a high adherence, and a long follow-up, we cannot exclude a lack of sufficient statistical power to detect a possible association, given the low event rate [49]. A second limitation consists of the lack of a specific genetic analysis for the identification of mutations in the *PCSK9* gene. However, given the relative rarity of the occurrence of loss- and gain-of-function mutations among the Caucasian population [50], the exclusion of these subjects from the analysis would have had a minimal and non-significant impact on the statistical analysis. Furthermore, although *PCSK9* levels were not assessed over time through the course of the trial, *PCSK9* levels are not expected to be affected by tamoxifen or fenretinide. Indeed, the *PCSK9* gene expression is regulated by sterol regulatory element binding proteins, which modulate the expression of *PCSK9* by binding the SRE element present in its promoter region [32,51]. Thus, *PCSK9* levels increase in response to treatment that reduces intracellular cholesterol synthesis, activating SREBP2. Thirdly, although no gold-standard assays are recognized for the evaluation of circulating *PCSK9*, the intra-assay and inter-assay coefficients of variability of the assay we used showed good reproducibility. This has been demonstrated by running this assay in our lab with approximately 8500 samples over the last few years [52,53]. Furthermore, the mean values we found in these women are in line with those we detected in women in early pregnancy [20].

## 5. Conclusions

Although *PCSK9* plasma levels did not appear to have a role as a circulating prognostic biomarker, at least in the context of early breast neoplastic events, its role in breast cancer progression in other settings should be explored, including the consideration of *PCSK9* breast tissue expression as well as its role in predicting the severity of the pathology.

## Figures and Tables

**Table 1:** Baseline anthropometric and biomarker results by risk category, expressed as medians and interquartile ranges.

Variable	5-Year Gail Risk ≥ 1.3% (*n* = 54)	IEN, T1 (*n* = 181)	*p*-Value
Age, y	46 (43, 49)	46 (42, 49)	0.868
BMI, kg/m^2^	22 (21, 25)	23 (21, 25)	0.524
Percentage mammographic density	49.9 (38.4, 61.1)	46.3 (35.3, 58.3)	0.312
*PCSK9*, ng/mL	206 (161–250)	207 (172–263)	0.554
17 β-estradiol, pg/mL	142.3 (94.4, 232.7)	121.7 (72.3, 188)	0.118
Total cholesterol, mg/dL	216 (198, 236)	208 (185, 234)	0.158
LDL-C, mg/dL	130.6 (109.8, 152.4)	122.3 (103.4, 145.5)	0.183
HDL-C, mg/dL	66.5 (61, 79)	69 (58, 78)	0.587
Non-HDL-C, mg/dL	144.5 (125, 168)	137 (118, 166)	0.272
Remnant cholesterol, mg/dL	14 (10.2, 18.6)	14.4 (11.6, 18.8)	0.261
Triglycerides, mg/dL	70 (51, 93)	72 (58, 94)	0.258
Leptin, ng/mL	10.01 (6.16, 16.0)	10.8 (7.3, 15.6)	0.497
Adiponectin, μg/mL	11.99 (8.62, 16.1)	9.8 (6.9, 13.5)	**0.006**
CRP, mg/L	0.07 (0.04, 0.18)	0.1 (0.04, 0.21)	0.192
Glucose, mg/dL	89 (83, 93)	88 (83, 94)	0.927
Insulin, μU/mL	12.32 (10.38, 15.12)	14.00 (10.72, 17.5)	**0.048**
HOMA-IR	2.65 (2.14, 3.26)	3.05 (2.31, 3.80)	**0.047**

IEN, Intraepithelial neoplasia; T1, small invasive cancer of favorable prognosis; BMI, body mass index; *PCSK9*, proprotein convertase subtilisin/kexin type 9; LDL-C, low-density lipoprotein cholesterol; HDL-C, high-density lipoprotein cholesterol; CRP, C-reactive protein; HOMA-IR, homeostatic model assessment of insulin resistance; data are expressed as medians and interquartile ranges. Values in bold represent significant differences.

**Table 2:** Baseline Spearman correlations between *PCSK9* and the other biomarkers.

	Univariate Spearman Analysis R Coefficient
BMI, kg/m^2^	0.080
Percentage mammographic density	−0.045
17 β-estradiol, pg/mL	−0.294
Total cholesterol, mg/dL	0.277
LDL-C, mg/dL	0.217
HDL-C, mg/dL	0.037
Non-HDL-C, mg/dL	0.256
Remnant cholesterol, mg/dL	0.237
Triglycerides, mg/dL	0.238
Leptin, ng/mL	0.171
Adiponectin, μg/mL	0.003
CRP, mg/L	0.166
Glucose, mg/dL	0.164
Insulin, μU/mL	0.066
HOMA-IR	0.092

*PCSK9*, proprotein convertase subtilisin/kexin type 9; BMI, body mass index; LDL-C, low-density lipoprotein cholesterol; HDL-C, high-density lipoprotein cholesterol; CRP, C-reactive protein; HOMA-IR, homeostatic model assessment of insulin resistance.

## Data Availability

Deidentified participant data underlying this article may be shared upon reasonable request to the corresponding author. A specific purpose for the data request is required due to privacy or ethical restrictions in force during the study conduction.
